# Trends, Prevalence of Bradyarrhythmia and Pacemaker Implantation in Patients with Parkinson’s Disease

**DOI:** 10.3390/jcdd12070252

**Published:** 2025-06-30

**Authors:** Tochukwu Nzeako, Olayemi Adeniran, Shoshanah Kahn, Neil Wimmer

**Affiliations:** 1 Department of Internal Medicine, Christiana Care Hospital, Newark, DE 19718, USA; 2 Department of Cardiovascular Diseases, SUNY Downstate University Hospital, Brooklyn, NY 11203, USA; olayemi.adeniran@downstate.edu; 3 Department of Cardiology, Northwell Health, Long Island, NY 11542, USA; 4 Department of Cardiovascular Diseases, Christiana Care Hospital, Newark, DE 19718, USA

**Keywords:** bradyarrhythmia, Parkinson’s disease, predictors, pacemaker implantation

## Abstract

Bradyarrhythmia is associated with an increased risk of falls, syncope, and sudden cardiac arrest in Parkinson’s disease (PD). However, studies investigating bradyarrhythmia in PA have been scarce. Therefore, we aimed to assess trends, prevalence, and risk factors of bradyarrhythmia and pacemaker implantation in PD patients. The National Inpatient Sample was utilized to identify patients’ data with primary and secondary diagnoses of Parkinson’s disease (PD) from 2016 to 2020. A total of 333,242 patients had a PD diagnosis; of these, 5092 (1.5%) had comorbid diagnoses of bradyarrhythmia. The prevalence of bradyarrhythmia in patients with PD was 351.9 per 10,000 hospitalizations (3.5%), with an increase from 291.9 to 463.8 per 10,000. However, the trends remained relatively stable. The overall prevalence of pacemaker implantation in patients with PD was 79.9 per 10,000 hospitalizations (0.8%). The overall trend of pacemaker implantation was stable in patients with PD. Age ≥ 65, male sex, and comorbidities (atrial fibrillation, coronary artery disease, heart failure, hypertension, liver failure, obesity, peripheral vascular disease, renal failure) were associated with a higher likelihood of bradyarrhythmia in patients with PD. This study’s findings revealed an increase in the prevalence of bradyarrhythmia. However, the prevalence of pacemaker implantation remained relatively stable over the study period.

## 1. Introduction

Parkinson’s disease (PD) is one of the most common neurodegenerative disorders [1], affecting more than one million persons in the United States [2]. It is characterized by the loss of dopaminergic neurons in the substantia nigra nuclei in the midbrain, resulting in movement abnormalities and other non-motor dysfunction [3,4]. The clinical manifestations of PD cause physical impairment and significant disability [5,6]

Autonomic nervous system dysfunction is a prominent non-motor component of PD, comprised of gastrointestinal, urinary, sexual, and cardiovascular dysregulation [7,8]. Cardiovascular dysautonomia is one of the hallmarks of autonomic dysfunction in PD [9]. Studies have attributed these manifestations to autonomic nerve denervation [10]. Moreover, patients with PD have an increased risk of developing structural and functional cardiovascular changes, such as ventricular hypertrophy, ischemic heart disease, heart failure, and arrhythmias [11]. Epidemiological studies have documented some of the common cardiovascular autonomic dysfunctions observed in PD, including orthostatic hypotension, supine hypertension, nocturnal hypertension, labile blood pressure, and heart rate variability [12]. Also, sympathetic nerve denervation in PD can cause sinus node dysfunction and blocks [13,14]. However, PD studies on bradyarrhythmia in PD are lacking.

Cardiovascular dysautonomia affecting the conduction system usually manifests as impaired function of the sinus node and cardiac conduction system and may cause bradyarrhythmia, including symptomatic bradycardia, atrioventricular (AV) block or sick sinus syndrome (SSS) [15]. However, no studies have assessed the prevalence and characteristics of bradyarrhythmia in patients with PD. In clinical practice, patients with bradyarrhythmia often present with nonspecific symptoms, which may include presyncope/syncope, dizziness, and lightheadedness requiring pacemaker implantation. Additionally, there are no studies available to date to assess the prevalence of pacemaker implantations in patients with PD.

Therefore, the aims of the present study were (1) to describe the trends, prevalence, and characteristics of bradyarrhythmia in patients with PD, (2) to assess the trends and prevalence of pacemaker implantations in patients with PD, (3) to investigate the predictors of bradyarrhythmia in patients with PD, and (4) to examine the prevalence of pacemaker complications in patients with PD.

## 2. Materials and Methods

### 2.1. Data Source and Study Population

The study utilized the National Inpatient Sample (NIS) database, compiled by the Agency for Healthcare Research and Quality (AHRQ), USA. The NIS database is the largest inpatient dataset in the US and is validated to provide reliable estimates of hospitalizations. It provides a nationally representative sample of approximately 20 percent of all discharges from US community hospitals [16]. It contains diagnoses and procedures coded using the International Classification of Diseases, 10th revision (ICD-10-CM). As a de-identified database, our study did not require the Institutional Review Board’s approval.

We conducted a retrospective cohort study of all adult hospitalizations from 1 January 2016 to 31 December 2020, with the primary and secondary diagnoses of PD, using ICD-10-CM codes, G20x. We further identified patients who had bradyarrhythmia. Bradyarrhythmia was defined as abnormalities in the heart rate or rhythm resulting in a pathologically slow ventricular response, which included sick sinus syndrome (SSS) and atrioventricular block (AVB). These were identified using ICD codes SSS (I495) and AVB (I440, I441, I442, I443, I4430, I4439). We excluded patients aged < 18 and those with missing data on age, sex, race, and in-hospital mortality. We also excluded patients transferred to another acute-care facility to reduce duplication. Furthermore, we extracted patient demographics, including age, sex (male and female), race/ethnicity (Whites, Blacks, Hispanics, Asians, and other races), and comorbidities (hypertension, diabetes, heart failure, hyperlipidemia, coronary artery disease, chronic lung disease, liver failure, renal failure, peripheral vascular disease, cancers, obesity, atrial fibrillation, and prior stroke, prior myocardial infarction (MI), prior percutaneous coronary intervention (PCIP, and prior coronary artery bypass surgery (CABG)). A list of ICD-10 codes used to identify these comorbidities is included in the supporting information Table S1 in the Supplementary Materials.

### 2.2. Study Outcomes

Our primary outcomes of interest included the trends and prevalence of bradyarrhythmia in patients with PD. Secondary outcomes of interest included the prevalence of pacemaker implantation in PD, predictors of bradyarrhythmia in PD patients, and complications of pacemaker implantation in PD, such as cardiac tamponade, hemothorax, pneumothorax, sepsis, and venous thromboembolism. The supporting information includes a list of ICD-10 diagnoses and procedure codes for identifying these in-hospital outcomes (see Table S1 in Supplementary Materials).

### 2.3. Statistical Analysis

To derive the prevalence of PD, we utilized the weighted estimates through survey trend weights. The prevalence of PD was calculated using the number of PD hospitalizations divided by the total number of hospitalizations each year per 10,000. Similar trends of PD hospitalizations were estimated for age (<65 and >65), sex (male and female), and race/ethnicity (Whites, Blacks, Hispanics, Asians, and others). Furthermore, trends in in-hospital outcomes were calculated as the total number of events per 10,000 hospitalizations. Descriptive data are shown in percentages, mean, and standard deviation, and tested with the chi-square and *t*-test for categorical and continuous variables. Temporal trends in bradyarrhythmia prevalence, baseline characteristics, pacemaker prevalence, and associated complications were measured by the average annual percentage change (AAPC), with 95% confidence intervals (CIs), using joinpoint regression models. The AAPC showed either a significant increase (positive change) or a decrease (negative change) if different from zero at the alpha 0.05 level. On the other hand, AAPC is deemed stable if there is no observed significant increase or decrease during the study period.

We assessed potential risk factors of bradyarrhythmia in patients with PD using a multivariable logistic regression model adjusted for age, sex, race or ethnicity, and comorbidities, including hypertension, diabetes, heart failure, hyperlipidemia, coronary artery disease, chronic lung disease, liver failure, renal failure, cancers, obesity, atrial fibrillation, and prior history of stroke, myocardial infarction, percutaneous coronary intervention, and coronary artery bypass surgery. A two-tailed *p* < 0.05 was considered statistically significant. Data manipulation and statistical analyses were performed using SAS 9.4 software (SAS Institute Inc., Cary, NC, USA). Joinpoint regression was conducted using Joinpoint software version 4.5.0.1 (Bethesda, MD, USA)

## 3. Results

### 3.1. Baseline Characteristics of the Study Population

Between 1 January 2016 and 31 December 2020, there were 344,194 patients with primary and secondary diagnoses of Parkinson’s disease (PD), accounting for about 0.1% of hospitalizations in the study period. After applying the exclusion criteria, 333,242 patients had a PD diagnosis. Of these, 5092 (1.5%) had comorbid diagnoses of bradyarrhythmia, and 328,150 (98.5%) had no bradyarrhythmia. Overall, patients with PD had a mean age of 76.5 years; 58.7% were males, and 80.1% were White. Patients with PD who had bradyarrhythmia were then compared to those without (Table 1). Compared to those without bradyarrhythmia, PD patients with bradyarrhythmia tended to be older (80.8 (7.4) vs. 76.4 (9.6), *p* < 0.0001), males (63.3% vs. 58.6%), and had more comorbidities, including hypertension (84.0% vs. 74.2%, *p* < 0.001), hyperlipidemia (56.9% vs. 46.5%, *p* < 0.001), diabetes (34.7% vs. 33.3%, *p* < 0.001), coronary artery disease (46.9% vs. 28.8%, *p* < 0.001), heart failure (45.1% vs. 24.1%, *p* < 0.001), atrial fibrillation (65.3% vs. 25.0%, *p* < 0.001), chronic lung disease (25.0% vs. 23.3%, *p* = 0.0060), liver failure (5.2% vs. 4.4%, *p* = 0.0164), peripheral vascular disease (14.1% vs. 8.4%, *p* < 0.001), renal failure (33.0% vs. 22.7%, *p* < 0.001), prior coronary artery bypass graft (CABG) (10.7% vs. 6.8%, *p* < 0.001), prior myocardial infarction(MI) (10.0% vs. 7.0%, *p* < 0.001), and prior percutaneous coronary intervention (PCI) (1.1% vs. 0.7%, *p* = 0.0034). Obesity and prior stroke showed no difference between the two cohorts (Table 1).

### 3.2. Prevalence and Trends of Bradyarrhythmia in Patients with PD

There were 5092 (1.5%) of PD patients with bradyarrhythmia. The overall prevalence of bradyarrhythmia in patients with PD was 351.9 per 10,000 hospitalizations (3.5%). According to types, sick sinus syndrome constituted the majority of bradyarrhythmia, 152.8 per 10,000 (1.5%); first-degree AVB, 95.9 per 10,000 (0.9%); second-degree AVB, 40.1 per 10,000 (0.4%), complete AVB, 50.5 per 10,000 (0.5%), and other AVB, 32.3 per 10,000 (0.3%) Table 2. The prevalence of bradyarrhythmia increased from 291.9 to 463.8 per 10,000 from 2016 to 2020. However, the trends remained stable (AAPC 12.5%, CI −0.2% to 26.8%; *p* = 0.01). Sick sinus syndrome increased from 129.3 to 221.8 per 10,000. However, the trend remained relatively stable (AAPC 14.8%: −9.8% to 46.1%; *p* = 0.07). The trends of other types of bradyarrhythmia increased significantly in the study period: first-degree AVB from 84.2 to 111.4 per 10,000 (AAPC 7.8%; 4.1% to 11.5%; *p* = 0.01); second-degree AVB from 36.7 to 45.6 per 10,000 (AAPC 6.4%; 0.9% to 12.3%; *p* = 0.02); complete AVB also increased from 44.8 to 61.0 per 10,000 (AAPC 8.5%; 2.3% to 15.1%; *p* = 0.01); and other AVB increased from 12.6 to 48.0 per 10,000 (AAPC 34.4%; 12.3% to 60.1%; *p* = 0.02) Table 2.

### 3.3. Prevalence and Trends of Pacemaker Implantation and Related Complications in Patients with PD

The overall prevalence of pacemaker implantation in patients with PD was 79.9 per 10,000 hospitalizations (0.8%). The overall trends of pacemaker implantation were stable in patients with PD during 2016–2020 (AAPC −0.9%; −4.1% to 2.3%; *p* = 0.66), Table 2. There were stable trends of pacemaker types during the study period: single chamber pacemaker (AAPC 4.0%; −10.6% to 20.7%; *p* = 0.45); dual chamber pacemaker (AAPC 1.3%; −4.9% to 7.8% *p* = 0.45); and biventricular pacemaker (AAPC 15.9%; −13.3% to 54.6%; *p* = 0.10). The prevalence of pacemaker complications in patients with PD included cardiac tamponade 3.8 per 10,000 (0.03%), hemothorax 28.5 per 10,000 (0.3%), pneumothorax 31.8 per 10,000 (0.3%), sepsis 1490.1 per 10,000 (14.9%), and venous thromboembolism 252.4 per 10,000 (2.5%). The trends of pacemaker implantation complications showed a stable increase in the study period for cardiac tamponade (AAPC 6.2%; −3.4% to 16.6%; *p* = 0.13), hemothorax (AAPC 17.9%; −2.6% to 42.1%; *p* = 0.06), pneumothorax (AAPC 10.4%; −4.6% to 27.5%; *p* = 0.08), and venous thromboembolism (AAPC 5.9%; −1.6% to 14.1%; *p* = 0.02). On the other hand, sepsis complications increased significantly from 1359.2 to 1659.1 per 10,000 (AAPC 4.3%; 1.4% to 7.2%; *p* = 0.02), Table 2.

### 3.4. Multivariate Analysis of Patient Variables Associated with Bradyarrhythmia

Table 3 presents patients’ characteristics and their effects in the multivariable model. Age ≥ 65 (Odds ratio (OR): 2.10; confidence interval (CI): 1.92–2.31), male sex (OR: 1.33; CI: 1.27–1.38), presence of atrial fibrillation (OR: 2.35; CI: 2.26–2.45), coronary artery disease (OR: 1.42; CI: 1.36–1.48), heart failure (OR: 1.47; CI: 1.40–1.53), hyperlipidemia (OR: 1.23; CI: 1.18–1.28), hypertension (OR: 1.31; CI: 1.24–1.38), liver failure (OR: 1.32; CI: 1.22–1.42), obesity (OR: 1.17; CI: 1.10–1.24), peripheral vascular disease (OR: 1.32; CI: 1.24–1.39), and renal failure (OR: 1.26; CI: 1.21–1.32) were associated with a higher likelihood of bradyarrhythmia in patients with PD. On the other hand, the presence of cancer (OR: 0.89; CI: 0.82–0.96) and chronic lung disease (OR: 0.84; CI: 0.80–0.88) showed a lower likelihood of bradyarrhythmia.

## 4. Discussion

The significant findings of this study were as follows: (1) The prevalence of bradyarrhythmia in patients with PD was 3.5%, with a stable increase from 2016 to 2020. Bradyarrhythmia was more prominent among older patients (mean age 80.8), males 63.3%, and the presence of multiple comorbidities. While SSS showed a stable increase, first-degree, second-degree, and complete AVB increased significantly in the study period. (2) Age ≥ 65, male sex, comorbidities (atrial fibrillation, coronary artery disease, heart failure, hypertension, liver failure, obesity, peripheral vascular disease, renal failure) were associated with a higher likelihood of bradyarrhythmia in patients with PD. (3) The prevalence of pacemaker implantation in patients with PD was 0.8%, with stable trends throughout the entire period. Also, the trends of pacemaker-related complications were stable during the study years, except for sepsis, which showed a significant increase. Our study is the first to investigate the prevalence of bradyarrhythmia in patients with PD in the US population.

A few prior studies have assessed the prevalence of bradyarrhythmia and pacemaker implantation in patients with PD. Most current information is driven by case reports and single-observation studies. The first report of bradyarrhythmia in PD was published in Japan. It was a case report that described a sick sinus syndrome (SSS) in a patient with confirmed PD and eventual pacemaker implantation [17]. The SSS was a result of autonomic failure, a common symptom in patients with PD. Another case report was a patient with PD who experienced persistent fainting spells; sick sinus syndrome was diagnosed, and a permanent cardiac pacemaker was implanted [18]. An observational study that explored the prevalence of bradyarrhythmia in patients with Lewy body disease, including PD, found a high prevalence of 5.2 percent [19].

There are several proposed reasons for the increasing bradyarrhythmia and subsequent pacemaker implantation in patients with PD. First, patients with PD are older and are at higher risk of developing conduction defects that predispose to bradyarrhythmia. In our study, PD patients with bradyarrhythmia had a mean age of 80.8 years. Prior studies have shown that sick sinus syndrome increases with age [20]. Sinus node dysfunction has a prevalence of about 1 per 1000 person-years in the United States, with a significant increase after age ≥ 65 [21]. Older age is associated with atrial stretching and remodeling that eventually affects the sinus node and other conduction systems of the heart, which might predispose to the development of bradyarrhythmia [22,23,24]. Also, it has been found that the pacemaker cells in the sinoatrial node decrease with age [25]. Second, male sex is another significant predictor of bradyarrhythmia in Parkinson’s disease in our study. Prior studies have shown that men generally exhibit longer PR intervals and atrioventricular refractory periods than women. This causes slower atrioventricular conduction and increased propensity towards bradyarrhythmia and blocks in men [26,27].

Another potential mechanism of bradyarrhythmia in PD is autonomic nervous system dysregulation. Cardiovascular dysautonomia represents a non-motor involvement in PD, which occurs early in disease progression [28,29]. With regard to the mechanisms responsible for these manifestations, it has been considered that α-synuclein and autonomic nerve denervation were the keystones of cardiac dysautonomia [30]. The accumulation of α-synuclein within the sympathetic ganglion, especially in the noradrenergic nerve fibers, is considerably correlated with the loss of cardiac noradrenaline and its neuronal storage [31].

Furthermore, patients with PD possess comorbidities, especially cardiac abnormalities, including cardiomyopathies, coronary artery disease, or heart failure. In our study, we found that comorbidities such as atrial fibrillation, coronary artery disease, heart failure, hyperlipidemia, hypertension, liver failure, peripheral vascular disease, and renal failure were associated with a higher likelihood of bradyarrhythmia in patients with PD. This finding is consistent with prior studies in PD that found an increased prevalence of cardiovascular disease and other risk factors in PD [32,33]. The presence of these cardiovascular causes an increased structural abnormality, including left ventricular hypertrophy, diastolic dysfunction, abnormalities of heart rhythm, and changes in the electrophysiological properties of the heart.

Our findings should be interpreted with caution, considering the following limitations. First, the NIS is an administrative hospital discharge database based on ICD coding, which can lead to the misclassification of cases. Second, our study identified the PD population using the ICD-10 code D571, which is susceptible to coding errors. The quality of the hospital discharge database has not been validated regarding the diagnosis of PD. Third, due to the retrospective nature of the database, there remains a potential for bias due to confounding factors despite adjustment for comorbidities. Despite these limitations, our study provides valuable and robust information on patients with PD, an area lacking in the United States. Despite these limitations, our study provides the first valuable and robust information on bradyarrhythmia in PD.

## 5. Conclusions

This study assessed the prevalence of bradyarrhythmia and pacemaker implantation in Parkinson’s disease. In addition, increasing comorbidities were associated with a higher likelihood of bradyarrhythmia in patients with Parkinson’s disease. While it remains hypothetical, mechanisms may be multifactorial with older age, cardiac dysautonomia, and fibrosis of the heart’s conduction systems as possible contributing factors. Further studies are critical to understand the increasing cases of bradyarrhythmia among patients diagnosed with Parkinson’s disease.

## Figures and Tables

**Table 1:** Baseline characteristics of patients with Parkinson’s Disease.

Characteristics	Total	Bradyarrhythmia	Non-Bradyarrhythmia	*p*-Value
**Unweighted, N**	333,242	5092 (1.5%)	328,150 (98.5%)	
**Weighted, N**	1,666,210	25,460	1,640,750	
**Age, mean (SD)**	76.5 (9.6)	80.8 (7.4)	76.4 (9.6)	<0.0001
**Female**	41.3	36.7	41.4	<0.0001
**Race**				
White	80.1	84.2	80.0	<0.0001
Black	7.0	4.8	7.0	
Hispanic	7.5	6.2	7.5	
Asians	2.5	2.8	2.5	
Others	2.8	2.0	2.8	
**Bradyarrhythmia**				
Sick sinus syndrome	1.5	43.4	0.0	
First-degree AVB	1.0	27.2	0.0	
Second-degree AVB	0.4	11.4	0.0	
Complete AVB	0.5	14.3	0.0	
Other AVB	0.3	9.2	0.0	
**Comorbidities**				
Diabetes mellitus	33.3	34.7	33.3	0.0313
Hypertension	74.4	84.0	74.2	<0.0001
Hyperlipidemia	46.6	56.9	46.5	<0.0001
Coronary artery disease	29.1	46.9	28.8	<0.0001
Heart failure	24.5	45.1	24.1	<0.0001
Chronic lung disease	23.3	25.0	23.3	0.0060
Renal failure	22.8	33.0	22.7	<0.0001
Liver failure	4.4	5.2	4.4	0.0164
Peripheral vascular disease	8.5	14.1	8.4	<0.0001
Cancer	6.6	5.2	6.6	<0.0001
Obesity	10.3	10.5	10.3	0.6135
Atrial fibrillation	25.6	65.3	25.0	<0.0001
History of stroke	2.9	2.8	2.9	0.8276
History of MI	7.0	10.0	7.0	<0.0001
History of PCI	0.7	1.1	0.7	0.0034
History of CABG	6.8	10.7	6.8	<0.0001
**Pacemakers**				
Single-chamber pacemaker	0.2	3.4	0.2	<0.0001
Dual-chamber pacemaker	0.7	17.9	0.4	<0.0001
Biventricular pacemaker	0.2	1.0	0.2	<0.0001
Any pacemaker	0.8	21.2	0.5	<0.0001

**Table 2:** Trends in the Clinical and Demographic Characteristics of the Study Cohort.

Characteristics	Total	2016	2017	2018	2019	2020	AAPC (95% CI)	*p* Value
**Parkinson’s disease Hospitalization per 10,000**	116.2	109.7	114.3	118.6	119.8	118.5	2.0 (−0.8 to 4.9)	0.05
**Age**								
<65	22.8	22.1	22.9	22.7	23.3	23.0	0.9 (−0.8 to 2.7)	0.17
≥65	237.2	229.1	233.9	241.2	240.0	241.6	1.3 (0.1 to 2.5)	0.04
**Sex**								
Male	159.6	152.0	157.1	163.0	164.3	161.3	1.6 (−1.2 to 4.5)	0.09
Female	83.8	79.2	83.0	85.6	86.3	85.1	1.8 (0.1 to 3.5)	0.08
**Race**								
White	138.9	129.7	136.2	141.6	143.1	144.5	2.7 (−0.1 to 5.5)	0.01
Black	53.1	50.7	52.0	54.0	55.0	53.9	1.8 (−1.4 to 5.1)	0.06
Hispanic	77.5	77.3	79.1	82.0	77.7	71.2	−1.8 (−8.5 to 5.3)	0.33
Asians	106.5	99.1	103.3	107.4	112.3	110.1	3.0 (0.9 to 5.0)	0.02
Others	89.6	84.5	87.4	87.3	95.6	93.1	2.9 (0.0 to 5.7)	0.04
**Bradyarrhythmia**	351.9	291.9	299.7	319.8	385.7	463.8	12.5 (−0.2 to 26.8)	0.01
Sick sinus syndrome	152.8	129.3	124.6	122.3	168.5	221.8	14.8 (−9.8 to 46.1)	0.07
First-degree AVB	95.9	84.2	83.7	98.6	101.1	111.4	7.8 (4.1 to 11.5)	0.01
Second-degree AVB	40.1	36.7	36.4	37.7	44.1	45.6	6.4 (0.9 to 12.3)	0.02
Complete AVB	50.5	44.8	44.9	46.8	54.9	61.0	8.5 (2.3 to 15.1)	0.01
Other AVB	32.3	12.6	29.3	32.0	38.8	48.0	34.4 (12.3 to 60.1)	0.02
**Pacemakers**								
Any pacemaker	79.9	77.1	82.5	83.1	82.6	73.6	−0.9 (−4.1 to 2.3)	0.66
Single-chamber pacemaker	23.4	18.3	26.2	25.1	23.9	23.3	4.0 (−10.6 to 20.7)	0.45
Dual-chamber pacemaker	69.5	65.3	69.6	70.3	74.1	67.7	1.3 (−4.9 to 7.8)	0.45
Biventricular pacemaker	18.3	10.7	19.0	20.1	19.3	22.0	15.9 (−13.3 to 54.6)	0.10
**Complications**								
Cardiac tamponade	3.8	3.5	3.4	3.3	4.3	4.3	6.2 (−3.4 to 16.6)	0.13
Hemothorax	28.5	16.2	29.6	29.2	31.0	36.0	17.9 (−2.6 to 42.1)	0.06
Pneumothorax	31.8	22.4	32.0	33.8	34.8	35.2	10.4 (−4.6 to 27.5)	0.08
Sepsis	1490.1	1359.2	1455.6	1491.3	1485.9	1659.1	4.3 (1.4 to 7.2)	0.02
Venous thromboembolism	252.4	222.6	245.8	249.2	251.0	293.7	5.9 (−1.6 to 14.1)	0.02

AAPC. Average annual percent change, expressed in percentages; CI. Confidence interval; AVB. Atrioventricular block.

**Table 3:** Adjusted odds ratios for bradyarrhythmia in Parkinson’s disease.

Variables	Adjusted OR	95% CI	*p*-Value
Age ≥ 65 vs. <65	2.10	1.92–2.31	<0.0001
Male vs. Female	1.33	1.27–1.38	<0.0001
**Race/Ethnicity**			
Whites	Ref	Ref	Ref
Blacks	1.01	0.94–1.09	0.0803
Hispanics	0.88	0.81–0.95	0.0368
Asians	0.98	0.86–1.11	0.5818
Others	0.89	0.78–1.00	0.1872
**Comorbidities**			
Atrial fibrillation	2.35	2.26–2.45	<0.0001
Cancer	0.89	0.82–0.96	0.0021
Chronic lung disease	0.84	0.80–0.88	<0.0001
Coronary artery disease	1.42	1.36–1.48	<0.0001
Diabetes mellitus	0.97	0.93–1.01	0.1075
Heart failure	1.47	1.40–1.53	<0.0001
Hyperlipidemia	1.23	1.18–1.28	<0.0001
Hypertension	1.31	1.24–1.38	<0.0001
Liver failure	1.32	1.22–1.42	<0.0001
Obesity	1.17	1.10–1.24	<0.0001
Peripheral vascular disease	1.32	1.24–1.39	<0.0001
Prior CABG	0.93	0.87–0.99	0.0187
Prior MI	0.96	0.90–1.03	0.2529
Prior PCI	0.94	0.78–1.14	0.5276
Prior Stroke	1.08	0.98–1.20	0.1385
Renal failure	1.26	1.21–1.32	<0.0001

OR. Odds ratio; CI. Confidence interval.

## Data Availability

The data used in this study were obtained from the National Inpatient Sample (NIS), which is part of the Healthcare Cost and Utilization Project (HCUP) maintained by the Agency for Healthcare Research and Quality (AHRQ). The NIS dataset is available for purchase and access through HCUP’s online database.

## Supplementary Materials

The following supporting information can be downloaded at: https://www.mdpi.com/article/10.3390/jcdd12070252/s1, Table S1: Description of codes for the data analysis.
