# Supplementary material for: Trends, Prevalence of Bradyarrhythmia and Pacemaker Implantation in Patients with Parkinson’s Disease
Source: J Cardiovasc Dev Dis. 2025 Jun 30;12(7):252. doi: 10.3390/jcdd12070252 (PMC12295930; doi:10.3390/jcdd12070252)
Supplement: Supplementary file 1 [file jcdd-12-00252-s001.zip › jcdd-3619249-supplementary.pdf]

Table S1. Description of codes for the data analysis.

Variables	ICD–10 codes
**Parkinson's disease**	G20x
**Bradyarrhythmia**	I495, I440, I441, I442, I443, I4430, I4439
**Sick sinus syndrome**	I495
**First-degree atrioventricular block**	I440
**Second-degree atrioventricular block**	I441
**Complete atrioventricular block**	I442
**Other atrioventricular blocks**	I443, I4430, I4439
**Single-chamber pacemakers**	0JH604Z,0JH605Z,0JH634Z,0JH635Z
**Dual-chamber pacemakers**	0JH606Z,0JH636Z
**Biventricular pacemakers**	0JH607Z,0JH637Z
**Diabetes Mellitus**	E08x – E11x; E13x; O24x; P702
**Hypertension**	I10-I12.0x, I129x, I13x; I15.2x, I15.8x, I15.9x, I16.0x, I16.1x, I16.9x, I67.4x, O10-O11x; O16x;
**Hyperlipidemia**	E78x
**Coronary artery disease**	I25x
**Heart failure**	I50.0x, I110, I130, I132
**Obesity**	E66x, O99x, Z68, R93.9
**Chronic kidney disease**	N18-N19x; Z40; Z91.115x, Z99.2
**Chronic lung disease**	J40-J45x; J47x; J60-J64x; J66-J67x; J68.4x
**Chronic liver disease**	B18x; I85x; K70x; K72-K76x; Z94
**Peripheral vascular disease**	A5200, A5201, A5202, A5209, I70x
**Cancer**	C00x-C34x, C37x-C79x, C7A, C7Bx, C80x-C86x, C88x, C90x-C96x, D00x-D09x, D469, D47Z9, E3121, E3122, E3123
**Obesity**	Z683x, Z684x
**Atrial fibrillation**	I48x
**History of stroke**	I63x,
**History of myocardial infarction**	I252
**History of percutaneous coronary intervention**	Z9861
**History of coronary artery bypass surgery**	Z951
**Cardiac tamponade**	I314
**Hemothorax**	J942, S271XXA, S272XXA
**Pneumothorax**	J95811, S270XXA
**Sepsis**	A40x, A41x, R6520
**Venous thromboembolism**	I824x, I26x
